# IgA Levels among Type 2 Diabetic and Non-Diabetic Patients with Periodontitis: A Prospective Clinical Study

**DOI:** 10.1055/s-0042-1755616

**Published:** 2022-09-27

**Authors:** Pooja Palwankar, Saumya Jain, Ruchi Pandey, Shakila Mahesh

**Affiliations:** 1 Department of Periodontology, Manav Rachna Dental College FDS, MRIIRS Faridabad, Haryana, India; 2 Department of Microbiology, Manav Rachna Dental College FDS, MRIIRS Faridabad, Haryana, India

**Keywords:** Enzyme linked immunosorbent assay, Immunoglobulin A, nonsurgical periodontal therapy, gingival crevicular fluid, diabetes mellitus, diabetes biomarkers

## Abstract

**Objectives** To estimate gingival crevicular immunoglobulin A(IgA) using enzyme-linked immunosorbent assay (ELISA) among type II diabetic patients with periodontitis.

**Materials and Methods** A non-randomized study was done of 40 periodontitis subjects with a mean age of 50 years and were recruited into two groups, Group A (Type II controlled diabetics with HbA1c < 7%) and Group B (non-diabetics with HbA1c between 4 and 6%). Both the groups underwent nonsurgical periodontal therapy (NSPT). The clinical parameters were recorded at baseline, 1, and 3 months. GCF sample was collected for the estimation of crevicular IgA at baseline and at 3 months.

**Statistical Analysis** Results were analyzed using parametric tests paired *t* -test and Student's *t* -test for every assessment point. The level of significance was set at *p* < 0.05.

**Results** Difference in IgA levels and clinical parameters was seen between diabetic and non-diabetic groups, which was statistically significant.

**Conclusion** Changes in crevicular IgA levels in patients with diabetic periodontitis can be used as a novel biomarker in assessing the inflammatory status.

## Introduction

The chronic inflammatory disease that results in the inflammation of supporting structures of the teeth is known as periodontitis. 1 2 3 IgA antibody is present at mucosal sites and has a vital part in mucosa defense. There are multifaceted non-inflammatory, anti-inflammatory, and proinflammatory functions shown by IgA. 4 Increased periodontal disease activity is seen as one of the indicators in GCF by IgA. 5 Periodontitis and diabetes have been bidirectional and are assessed through the pathogenicity; increase in clinical attachment loss is associated with diabetes and pre-diabetics. 6 7 This study was done to estimate and compare the IgA levels in GCF using ELISA among type II diabetics and non-diabetics patients with periodontitis. Crevicular IgA could be a promising pro-inflammatory biomarker to assess the exacerbation of periodontal disease activity and impaired immune response in diabetic subjects with periodontitis.

## Materials and Methods

### Study Groups and Design

A methodical prospective clinical study was conducted on type II controlled diabetic and nondiabetic subjects with periodontitis of age group 35 to 65 years, as per the approval from institutional ethics committee, and was registered CTRI/2020/04/024712. A written participant information sheet was given and a participant informed consent in writing was drawn from all participants. This study enlisted 40 subjects (320 sites) of both sexes on the basis of HbA1c values, mild-to-moderate periodontitis (as per the 1999 classification by AAP.) pocket of 4 to 6 mm in at least two mesial sites in four quadrants. Group A consisted of type II diabetics with HbA1c < 7% and Group B consisted of non-diabetics with HbA1c between 4 and 6%. Both the groups underwent nonsurgical periodontal therapy. Participants who had undergone oral prophylaxis or were on antibiotics for last 3 months, pregnant or lactating mothers, or using any form of tobacco were not included in the study.

**Fig. 1:**
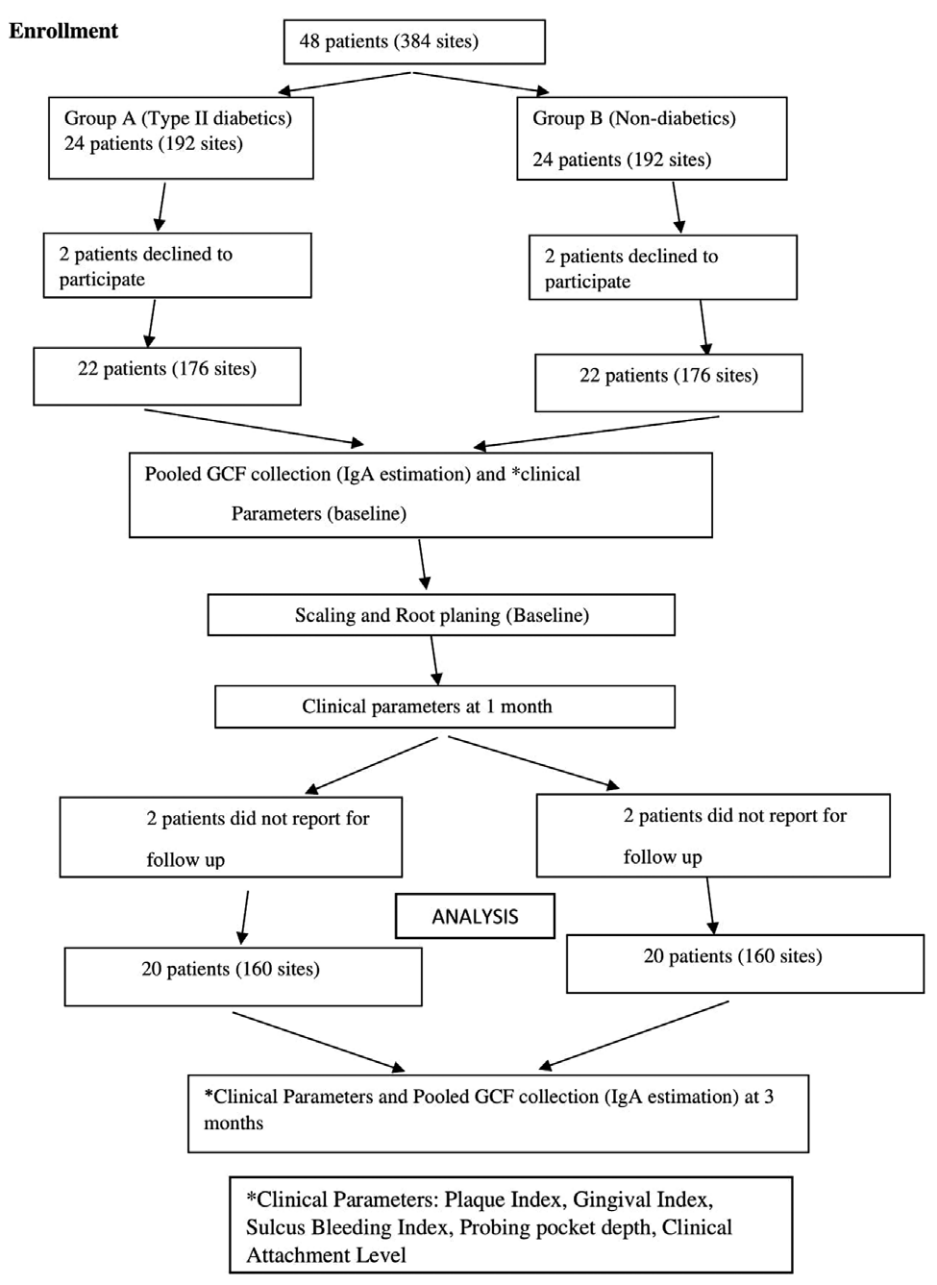
Consort Flow Chart.

Plaque (PI), gingival (GI) and sulcus bleeding (SBI), (Turesky–Gilmore–Glickman's modification of the Quigley and Hein, Loe and Silness, Muhlemann H.R and Son, S. probing pocket depth (PPD), and clinical attachment level (CAL) were recorded at initial (baseline) visit, 1 and 3 months. Oral hygiene instructions were reinforced at every recall visits ( Fig. 1 ).

### Collection of GCF and IgA Estimation

The pooled GCF sample was collected by 5 μL calibrated capillary micropipettes, from the eight mesial sites for each participant (two mesial sites per quadrant) and hoarded in polypropylene tubes at −80°C by the principal investigator. Each tube was coded by the observer to avoid the bias in formulating the biochemical analysis. The biochemical analyst was unaware of groups. The tubes were uncoded to formulate the results. The analysis was done with ELISA reader of BeneSphera ^®^ using the ElabScience ^®^ kit using sandwich-enzyme-linked immunosorbent assay (ELISA) principal. 8

### Statistical Analysis

The data were tabulated and collected in a Microsoft excel sheet and was subjected to statistical analysis using SPSS version 22.0 (SPSS Inc, Chicago, USA). Means and standard deviations calculated for each group were further assessed for statistical significance using parametric tests.

## Results

### Clinical Parameters

The changes in the plaque index were seen from baseline to 3 months in both the groups. Significant changes were noted at all intervals ( *p* < 0.05). The mean plaque value showed changes in both the groups from baseline to 1 month (0.576), baseline to 3 months (0.936), and 1 month to 3 months (0.326). Gingival index, pocket depth, sulcus bleeding index in the diabetic group and non-diabetic groups showed significant changes from initial visit to 3 months. The gingival index mean value changes in both the groups were 0.108, 0.474, and 0.435 from baseline to 1 month, baseline to 3 months, and 1 month to 3 months, respectively. The mean value changes in sulcus bleeding index from baseline to 1 month, baseline to 3 months, and 1 month to 3 months were 0.057, 0.023, 0.694, pocket depth was 0.324, 0.574, 1.000, and clinical attachment level was 0.154, 0.154, 0.180, respectively ( Tables 1 and 2 ).

**Table 1:** Intergroup and intragroup changes in PI, GI, and SBI at various intervals

Clinical parameters	Change from Baseline to 1 month		Change from 1 to 3 months		Change from baseline to 3 months	
Plaque index	Mean	SD	Mean	SD	Mean	SD
Type II diabetic	0.532	0.30	0.69	0.29	1.22	0.21
Nondiabetic	0.477	0.31	0.73	0.23	1.21	0.29
Type II diabetic v/s nondiabetic ( *p* -value)	0.576#		0.326#		0.936#	
Change in type II diabetic ( *p* -value)	0.0001*		0.0001*		0.0001*	
Change in nondiabetic ( *p* -value)	0.0001*		0.0001*		0.0001*	
Gingival index	Mean	SD	Mean	SD	Mean	SD
Type II diabetic	0.23	0.12	0.23	0.08	0.45	0.14
Nondiabetic	0.30	0.17	0.19	0.19	0.49	0.19
Type II diabetic v/s non-diabetic ( *p* -value)	0.108#		0.435#		0.474#	
Change in type II diabetic ( *p* -value)	0.0001*		0.0001*		0.0001*	
Change in nondiabetic ( *p* -value)	0.0001*		0.0001*		0.0001*	
Sulcus bleeding index	Mean	SD	Mean	SD	Mean	SD
Type II diabetic	0.38	0.15	0.27	0.09	0.65	0.15
Nondiabetic	0.28	0.15	0.26	0.11	0.54	0.13
Type II diabetic v/s nondiabetic ( *p* -value)	0.057#		0.694#		0.023#	
Change in type II diabetic ( *p* -value)	0.0001*		0.0001*		0.0001*	
Change in nondiabetic ( *p* -value)	0.0001*		0.0001*		0.0001*	

Abbreviations: *p* , probability; SD, standard deviation.

* *p* -Value significant at <0.05, # non-significant.

**Table 2:** Comparison of pocket depth and clinical attachment level at various time intervals

Clinical parameters	Change from Baseline to 1 month		Change from 1 to 3 months		Change from baseline to 3 months	
Probing pocket depth	Mean	SD	Mean	SD	Mean	SD
Type II diabetic	0.95	0.19	0.05	0.14	1.00	0.23
Nondiabetic	1.00	0.26	0.04	0.23	1.04	0.33
Type II diabetic v/s nondiabetic ( *p-* value)	0.324#		1.000#		0.574#	
Change in type II diabetic ( *p* -value)	0.0001*		0.083#		0.0001*	
Change in nondiabetic ( *p* -value)	0.0001*		0.064#		0.0001*	
Clinical attachment level	Mean	SD	Mean	SD	Mean	SD
Type II diabetic	0.938	0.44	0.006	0.08	0.94	0.45
Nondiabetic	0.87	0.62	0.02	0.22	0.90	0.63
Type II diabetic v/s nondiabetic ( *p-* value)	0.154#		0.180#		0.154#	
Change in type II diabetic ( *p* -value)	0.0001*		0.319#		0.0001*	
Change in nondiabetic ( *p-* value)	0.0001*		0.158#		0.0001*	

Abbreviations: *p* , probability; SD, standard deviation.

* *p* -Value significant at <0.05, # non-significant.

### Biochemical Parameters

Statistically significant differences were seen in crevicular IgA from baseline to 3 months (2.09 ± 1.23) for type II diabetics and 0.77 ± 0.45 for nondiabetics ( *p* < 0.05) ( Table 3 ).

**Table 3:** Comparison of changes in crevicular IgA at different time intervals

Groups Crevicular Immunoglobulin A	Change from Baseline to 3 months	
	Mean (ng/mL)	SD
Type II diabetic	2.09	1.23
Nondiabetic	0.77	0.45
Type II diabetic v/s nondiabetic ( *p* -value)	0.0001*	
Change in type II diabetic ( *p* -value)	0.0001*	
Change in nondiabetic ( *p* -value)	0.0001*	

Abbreviations: *p* , probability; SD, standard deviation.

* *p* -Value significant at < 0.05, # non-significant.

Mean crevicular IgA values at baseline for type II diabetics and non-diabetics were (4.25 ± 2.23) and (1.68 ± 0.88), respectively. The Mean HbA1c level at baseline for the type II diabetic group and nondiabetic group showed a positive correlation ( Table 4 ).

**Table 4:** Comparison of study groups with respect to crevicular IgA and HbA1c at baseline

Groups	Crevicular IgA at Baseline		HbA1c at Baseline	
	Mean (ng/mL)	SD	Mean	SD
Type II diabetic	4.25	2.23	6.60	0.26
Nondiabetic	1.68	0.88	5.54	0.18
*p* -Value	0.0001*		0.0001*	

Abbreviations: HbA1c, glycated hemoglobin; IgA, immunoglobulin A; p, probability; SD, standard deviation.

* *p* -Value significant at < 0.05, # non-significant.

## Discussion

Diabetes remains undiagnosed at sub-clinical levels due to asymptomatic trend, a potent biomarker is needed to evaluate the status of systemic diseases at the natal stage. 6 IgA present at mucosal surfaces is seen as the most potent antibody. This immunoglobulin is produced in excess quantity by than other antibodies. The IgA role is important in defense mechanism of host and pathogens. Immune complexes of IgA are seen to initiate the cells potentially via cross-linking at FcαRI (a member of Fc receptor immunoglobulin family), resulting in instigating responses, which are pro-inflammatory and thus eliminating the disease-causing microbes. 9

The intragroup comparison of scores of PI, GI, SBI, PPD, and CAL of two groups showed significant changes from base values to 1 month, which mainly occurred due to the conclusiveness of NSPT and maintenance of oral hygiene by the subjects during the estimated study period. Changes in scores from baseline to 3 months were also significant in both the groups. The above findings were attributed to the proper maintenance of standardized oral hygiene instructions during the estimated time period of the study following the treatment at the baseline. 10 11 12 13 14 15 16 17

On intergroup comparison, changes were not seen to be statistically significant in both study groups at various duration periods for all parameters clinically seen. 18

There was a reduction seen in the crevicular IgA level in both type II diabetic and nondiabetic groups from baseline to 3 months. In intragroup and intergroup comparisons, statistically, significant differences were seen amongst both the groups from baseline to 3 months. The reduction could be attributed to phase I therapy and reinforcement of oral hygiene instructions at every visit. In a similar study by Butchibabu et al, where salivary IgA was evaluated, it was reported that salivary IgA levels were increased with the periodontitis patients than healthy controls. 19

Levels of crevicular IgA in the present study were seen to be higher in type II diabetics than nondiabetics as diabetics with periodontitis are shown to have depressed chemotaxis of peripheral blood leukocytes and defect in phagocytosis. There was a correlation between crevicular IgA and HbA1c levels in both groups at baseline. This was evidenced by Awartani et al who stated poor diabetic control is associated with an elevation in IgG and IgA serum antibodies. 20

## Limitations

Microbiological analysis could have helped in identifying the specific periodontal pathogens affected in the study. Longer duration of study and postoperative assessment would have given a clear judgment and evaluation of the results. An increased probing pocket depth with severe loss of clinical attachment would have given a different result. Post treatment HbA1c levels at 3 months would have given the clear association with IgA level.

## Conclusion

A positive association was seen between HbA1c and levels of IgA. Levels of IgA were found to be elevated in type II diabetics than nondiabetics at baseline. Reduction was seen in IgA levels at the end of 3 months. It can be summarized within the confines of the study that IgA is a significant biomarker to ascertain the periodontal disease activity in diabetics. Randomized clinical studies involving larger sample size and longitudinal studies are recommended to further evaluate the link between crevicular IgA levels in diabetics with periodontitis.
